# Parasite Stress Predicts Offspring Sex Ratio

**DOI:** 10.1371/journal.pone.0046169

**Published:** 2012-09-26

**Authors:** Madhukar Shivajirao Dama

**Affiliations:** Institute of Wildlife Veterinary Research, Karnataka Veterinary, Animal and Fisheries Sciences University, Doddaluvara, Somavarpet taluk, Kodagu District, Karnataka, India; University of Turku, Finland

## Abstract

In this study, I predict that the global variation of offspring sex ratio might be influenced in part by the level of parasite stress. From an energetic standpoint, higher gestational costs of producing a male offspring could decrease male births in a population with limited resources. This implies that, any factor that limits the parental resources could be expected to favor female offspring production. Human sex ratio at birth (SRB) is believed to be influenced by numerous socioeconomic, biological, and environmental factors. Here, I test a prediction that parasite stress, by virtue of its effects on the general health condition, may limit the parental investment ability and therefore could influence the SRB at the population level. The statistical analysis supports this prediction, and show that the level of parasite stress has a significant inverse relation with population SRB across the world. Further, this relation is many-folds stronger than the association of SRB with other factors, like; polygyny, fertility, latitude, and son-preference. Hence, I propose that condition affecting ability of parasites (but not adaptive significance) could be a likely causal basis for the striking variation of SRB across populations.

## Introduction

Being larger in size, males require higher nutritional investment and are more vulnerable to variation in parental condition [1], [2]. Furthermore, males tend to have higher variance in reproductive success compared to females [3], [4]. Hence, according to the sex allocation theory of Trivers and Willard [5], it could be predicted that parents in good condition should bias investment towards male offspring to enhance future reproductive success. Conversely, parents in poor condition could enhance reproductive returns by biased investment in female offspring [5].

Human populations show a striking variation in offspring sex ratio at birth (SRB, defined as the ratio of male births to female births in a population). This global variation is believed to be an outcome of compound influence of staggering number of biological, social and environmental factors [6]–[8]. Although numerous studies have explored the effect of extra-genetic stimuli on temporal and spatial variation of SRB, only three empirical studies have attempted to explain the global variation in SRB. First, Barber (2004) tested the coital rate hypothesis proposed by James (1971), which predicts that more female conceptions result from intercourse around the time of ovulation, whereas more male conceptions result from inseminations occurring outside the peri-ovulatory period [9]. He reported that, across 148 countries, SRB correlated with the proportion of women using any form of contraception (r = 0.42), polygyny (r = −0.41), and total fertility (r = −0.60). Further, using multiple regression analysis; he showed that SRB correlated positively with wealth and mother’s age. Though the results were in line with the coital rate hypothesis, Barber (2004) suggested that the relationships may not be functional and may be influenced by other factors that could affect marriage system and fertility as well as SRB [10]. James suggest that the coital rate hypothesis depend on two well established phenomena i.e., the luteal hormone peak in the middle of the cycle, and the contemporaneous rise in the probability of male birth in the middle of the cycle [11]. Second, Navara (2009) predicted that latitudinal variation of climatic factors may create the difference in resource availability and consumer density between temperate and tropical regions, which could lead to cross-national variation of SRB [12]. She examined SRB in relation to latitude and associated climatic variables (LAT; principal component derived from latitude, average temperature and the annual day length) as well as with socioeconomic status (SS; principal component derived from average gross domestic product, unemployment rate and instability index). Using nested multiple logistic regression modeling; Navara (2009) showed that, across 202 countries, SRB was positively correlated with LAT, but not with SS. Navara concluded that tropical populations produce significantly more daughters compared to temperate and subarctic populations. The author offered little explanation of why this trend exists, and suggested that the latitudinal variation of SRB may represent the vestiges of prior selection based on factors such as day length and ambient temperature, which are believed to influence male fertility and SRB [13], [14]. Third, Dama (2011) predicted that the measures of parental condition may explain the variation in SRB across the world [15]. He found a positive correlation of SRB with population health indicators, and suggested that the cross-national differences in SRB may not necessarily be adaptive, and may be the outcome of reproductive constraints imposed by health-related factors [2], [16]. Considering the sexual dimorphism in cost of offspring production and reproductive success, positive correlation of SRB with health variables (which was stronger than total fertility, latitude and wealth) shows that the influence of parental condition on global variation of SRB is stronger than the influence of socioeconomic and geographical factors.

Parasitism is a key ecological factor that influences the condition of parents, and in effect, the potential fitness of their offspring [17]. Parasites adversely affects the body condition by feeding on host tissues, reducing the absorption of nutrients from the intestine, using host cellular machinery for their survival, and making host invest in defense mechanisms at the cost of other physiological processes [18]. In addition to accounting for 26% of mortality due to all causes around the world, parasites produce high level of morbidity, resulting in substantial deterioration of body condition [19]. Prevalence of disease causing human parasites is highest in tropics and becomes lesser with the increase in latitude. This distribution of parasite stress is driven by ecological factors that show latitude dependent trends [20]. Interestingly, empirical evidence suggests that parasites constrain the parental ability to invest in costlier offspring (sons) in birds [21].

I provide statistical evidence that parasites may have similar influence in humans, and constrain the ability of mothers to invest in male offspring, thereby producing a parasite stress dependent pattern of offspring sex ratio across the world.

## Materials and Methods

### Data

Sex ratio at birth for the year 2009 was taken from United States CIA (Central Intelligence) Agency [22]. A ratio of 1 indicate equal number of male and female births, whereas a ratio above or below 1 means more male or female births respectively. There are differences between the CIA estimates and numbers reported by the Census offices of Switzerland, Sweden, Norway, Ireland, India and Japan. However, these differences are minor and CIA data are accepted and widely used by cross-cultural researchers [10], [12], [23].

Disability-adjusted life years (DALY) lost due to parasitic diseases was used as a measure of parasite stress across nations [24]. One DALY owing to parasites equals one healthy year of life lost per one million people. This measure covers disability due to 28 important parasites across the world. Other measures of cross-national parasitic prevalence exists, however DALY will be more suitable for testing present hypothesis, as the estimates are available for most part of the world (n = 192) and directly reflect the effects of parasite in terms of physiological costs incurred by the affected individuals. Hence, DALY could be considered as a direct measure of deterioration of the condition owing to parasites. DALY owing to infectious diseases correlates strongly with other measures of parasitic diseases [25]. Apart from parasites, nutritional stress could be a major cause of cross-national differences in condition. The DALY owing to protein energy malnutrition, iodine deficiency, vitamin A deficiency and iron deficiency was used as an independent measure of nutrition stress. These variables were log transformed for normality. Henceforth, DALY owing to parasites and nutrition will be referred as parasite stress and nutritional stress respectively.

Contraceptive prevalence, which indicates the proportion of women of reproductive age who are using (or whose partner is using) a contraceptive method for the period 2005 to 2009, was obtained from United Nations [26].

Indicator of Polygyny for the year 2009 was obtained from Gender, Institutions and Development Database [27]. Polygyny is defined as men having multiple wives simultaneously. Countries were coded as 0 =  generally not accepted/polygyny is not legal in a country, 0.5 =  accepted by part of the population/polygyny is only legal for some people, or 1 =  generally accepted/polygyny is legal in a country.

Son preference is prevalent in many of the Asian countries and correlates positively with sex ratio at birth. Son preference (missing women) describes the difference between the number of women that should be alive (assuming no son preference) and the actual number of women in a country. All the countries are assigned values between 0 (no women are missing) and 1 accordingly. Values for prevalence of son preference for the year 2009 were obtained from Gender, Institutions and Development Database [27]. As son preference results in female selective abortions [28], it is necessary to adjust for the level of son preference in statistical analysis.

Total fertility estimates for the year 2008 were taken from World Bank [26]. Total fertility rate represents the number of children that would be born to a woman if she were to live to the end of her childbearing years and bear children in accordance with current age-specific fertility rates. The estimate includes all the children born dead or alive.

Gross national income per capita based on purchasing power parity (GNI, 2009) was used as a measure of wealth. GNI is the sum of value added by all resident producers plus any product taxes (less subsidies) not included in the valuation of output plus net receipts of primary income (compensation of employees and property income) from abroad. GNI, calculated in national currency, is usually converted to U.S. dollars at official exchange rates for comparisons across economies. GNI data was taken from the World Bank [26]. Values were log transformed for normality.

Mother age was calculated as mode estimated as the centre point of the five-year age block with the highest fertility in a country as explained by Barber (2004).

Latitude values for nations were obtained from the Central Intelligence Agency (CIA) World Factbook [22] and numerical values were used irrespective of direction.

As a measure of mortality level for each nation, health adjusted life expectancy AT BIRTH (HALE for the year 2007), estimated by WHO was used [29]. While, life expectancy at birth summarizes the mortality pattern that prevails across all age groups, HALE adds up expectation of life for different health states and measures average number of years that a person can expect to live in “full health” by taking into account years lived in less than full health due to disease and/or injury. These two measures reflect age-standardized summary of mortality in a population, however only HALE will be used in the present analysis as it is a more complete estimate of health than standard life-expectancy rates. Mortality rates at different stages of life were; infant mortality rate (2009), under-five mortality rate (2009), maternal mortality ratio (2008) and adult mortality rate (2008) were obtained from World Bank [26]. While infant mortality rate and maternal mortality ratio are actual number of deaths of infant (during the first year of life per 1000 live births in a given year) and mothers (per 100000 live births in a given year), under 5 mortality rate and adult mortality rate are the probabilities of dying before reaching the age of five and between the age of 15 to 65 respectively.

Descriptive properties of these raw variables are summarized in Table S1. It must be noted that SRB values were for the year 2009, whereas some factors were for 2009, and others were estimates for few years earlier to 2009. This discrepancy is owing to longer sampling frequency for most of the social variables. These differences are believed not to affect the statistical outcomes, as changes if any, in social factors are produced very gradually. Hence, it is an accepted norm to use data from nearest sampling year when data for the desired year are not available [10], [30].

### Estimation of Missing Data

The missing data were estimated by expectation maximization (EM), which is an iterative method for finding maximum likelihood estimates of parameters. The EM approach estimates unmeasured data and is based on iterating through two alternating steps [31]. In the expectation step, an appropriate value is calculated for the missing observation based on the available data and its distribution. In the maximization step, an appropriate value is calculated based on the current updated dataset. These two steps were alternated 100 times: such that after each expectation step a maximization step was followed. After the final iteration, theoretically the most accurate estimation of the missing values is reached, and these values were imputed into the dataset as a replacement for the missing observation. The imputed datasets were validated by calculating *t* values and effect sizes for the differences between the raw values and complete dataset.

### Data Reduction

To reduce the number of variables, principal component analysis was performed on the 5 mortality variables (Health adjusted life expectancy, Adult mortality rate, Maternal mortality ratio, Under-five mortality rate, and Infant mortality rate), after first confirming that the data were suitable for analysis. Principal components analysis (PCA) was followed by oblique rotation of the factors using Oblimin rotation (delta  = 0). The number of factors to be retained was guided by Kaiser’s criterion (eigenvalue >1), and inspection of the scree plot. Cronbach’s alpha was used to assess the internal consistency of the subscales identified from the PCA.

### Statistical Analysis

First, a multiple ridge regression model was constructed where all the independent factors were included as predictors of SRB. As many of the factors showed high collinearity (square root of variance inflation factor greater than 2.0) in ordinary least squares regression model, ridge regression model was employed [32]. Ridge regression artificially reduces correlation coefficient of each pair of variables by incorporating a ridge parameter to the diagonal of a correlation matrix of highly collinear independent variables, leading to reduced error variance of estimators. Based on this principle, ridge regression overcomes the collinearity problem [33]. Second, I used linear regression in an exploratory analysis to determine the relative weight of support for each of the ten variables as predictors for SRB; the goal of this analysis was to identify the potential factors for predicting the offspring sex ratio. Ten independent linear regression models were constructed, in order to identify the relative importance of weights from Akaike Information Criterion statistics (AIC) for each of the variables [34]. I computed AIC weights to compare these models. Following Burnham and Anderson [34] the best-fit model is that with the lowest AIC. As a rule of thumb, models with delta AIC (Δ_i_) within 2 units were considered to have substantial support, whereas models with Δ_i_ value of 3–7 and >10 are considered as less supportive and very unlikely, respectively. The ratio of AIC weights (w_i_/w_j_) for any two models is called an evidence ratio, and it quantifies the relative degree of support in the data for one model as compared to another [34].

Cross-cultural studies are criticized for exacerbation of the problem of non-independence (i.e. individual country, which is a unit of analysis, may not be necessarily truly independent statistical data-point). To overcome this problem, I have coded the countries by continents and included this variable in the regression models, to make each country an independent data-point [12]. All the statistical analyses were performed using SPSS 16.0.0 statistical software. Before accepting the final regression model, the residuals were confirmed to be homoscedastic (Breusch–Pagan test, p>0.05) and normally distributed (Ryan-Joiner’s test, p>0.05).

## Results

To supplement the visual analysis with statistics, table 1 shows t statistics, and Cohen’s d effect sizes for the estimation of missing data. The t and d values quantify the differences between the variables before and after imputation of the missing values. Very small values of t statistic and d validate the imputation method by suggesting that the mean and standard deviation of the datasets are not significantly altered by imputation.

**Table 1:** Independent samples t test for estimated data against missing data.

Variable	t	Degrees of Freedom	P	Cohen’s D
Parasite stress	0.3441	416	0.7309	0.034
Nutritional stress	0.5030	416	0.6153	0.049
Contraception	1.0076	401	0.3143	0.101
Polygyny	0.9555	343	0.3400	0.103
Son preference	1.4031	343	0.1615	0.152
Total fertility	0.1816	419	0.8560	0.018
Wealth	0.4303	403	0.6672	0.043
Mother age	0.1687	413	0.8661	0.017
Health adjusted life expectancy	0.6093	416	0.5427	0.059
Adult mortality rate	0.5742	411	0.5661	0.057
Maternal mortality ratio	0.7764	396	0.4380	0.078
Under-five mortality rate	0.4793	416	0.6320	0.047
Infant mortality rate	0.5030	416	0.6152	0.049

Principal Components Analysis of the 5 mortality items revealed one factor with eigenvalue exceeding one (4.53), accounting for 90.68% of the total variance. This factor was labeled “Health Factor”. Cronbach’s alpha reliability coefficient value of 0.7 indicated satisfactory internal consistency for the health factor.

**Figure 1:**
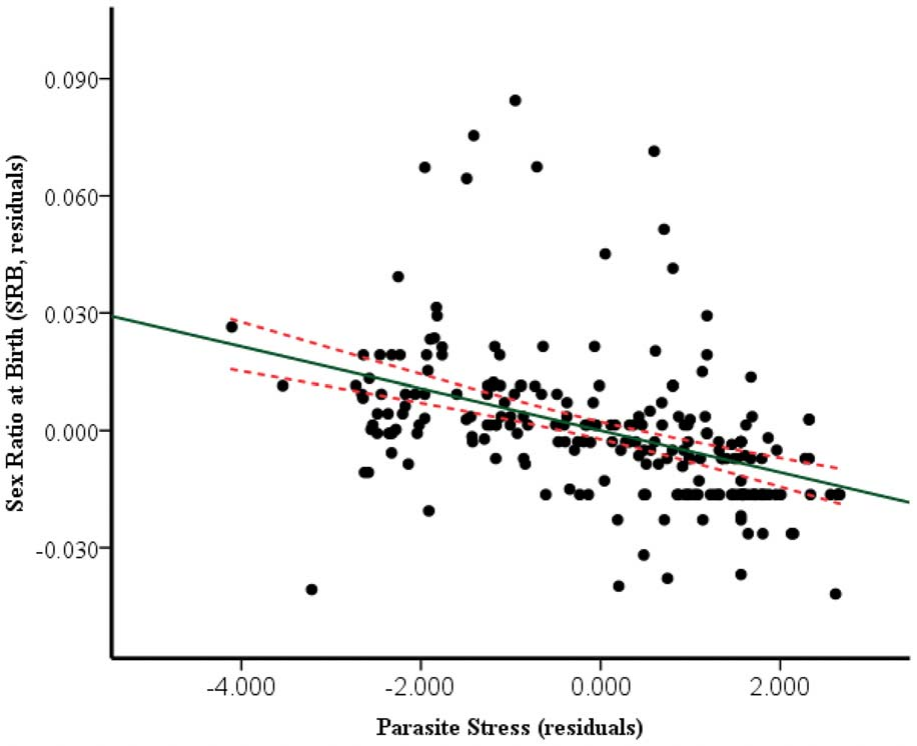
Predicted sex ratio at birth as a function of parasite stress across 226 countries. Solid line represents the fitted linear trend and dotted lines its 95% confidence intervals.

Ridge regression model showed a negative association of SRB with polygyny, total fertility, and parasite stress. This association suggests that population with higher rates of polygyny, higher fertility, and parasite stress produce significantly less number of male offspring. Among all the variables, son preference and latitude were positively associated with SRB (Table 2 and Figure 1).

**Table 2:** Ridge regression summary for predicting offspring sex ratio using test variables.

	Beta	Std.Err.	B	Std.Err.	t_(214)_	p-level
**Intercept**			1.089	0.024	44.50	0.000
**Parasite stress**	−0.262	0.097	−0.003	0.001	−2.71	0.007
**Son preference**	0.251	0.059	0.026	0.006	4.27	0.000
**Fertility**	−0.201	0.100	−0.003	0.001	−2.00	0.047
**Polygyny**	−0.150	0.074	−0.009	0.005	−2.03	0.043
**Latitude**	0.147	0.069	0.000	0.000	2.14	0.034
**Nutritional** **stress**	0.142	0.092	0.003	0.002	1.56	0.121
**Health factor**	−0.052	0.115	−0.001	0.002	−0.45	0.653
**Contraception**	−0.055	0.084	−0.000	0.000	−0.65	0.516
**Wealth**	−0.063	0.095	−0.001	0.002	−0.67	0.506
**Mother age**	−0.099	0.063	−0.001	0.000	−1.58	0.116
**Continent**	−0.001	0.062	−0.000	0.001	−0.02	0.987

Whole model: n = 226 countries, R^2^ = .32391749, F_(11,214)_ = 9.3209, p<0.00000.

AIC-based model selection showed that the best-fit model included only parasite stress, which alone explained 21.3% of SRB variation across the world (AICw  = 0.956, F = 30.21, P<0.001, R^2^ = 0.213; Table 3). Among the 10 factors, the model with parasite stress has a 95.68% probability of being the best model (AICw  = 0.956), whereas no other factor was found to offer substantial evidence similar to parasite stress (all nine models had Δi value of >7). The next most important variable was total fertility, with the AICw of 0.028 (Table 3), followed by health factor, latitude, son preference, contraception, polygyny, wealth, nutritional stress, and maternal age in that order. The evidence ratio indicated that the model with parasite stress is at least 33.56, 71.6, and 772.58 times more likely to predict SRB variation as compared to total fertility, health factor and latitude respectively.

**Table 3:** The best-fit models among the ten variables, based on AIC-based model selection.

Model	F	R^2^	P	AIC	Δi	*Wi*	Evidence ratio
Parasitestress	30.20	0.213	<0.001	−1838.04	0.00	0.956882	–
Totalfertility	25.86	0.188	<0.001	−1831.02	7.03	0.028510	33.56253208
Healthfactor	25.94	0.183	<0.001	−1829.5	8.54	0.013364	71.60023275
Latitude	22.10	0.165	<0.001	−1824.74	13.30	0.001239	772.5870843
Sonpreference	15.05	0.119	<0.001	−1812.48	25.56	0.000003	354832.7544
Contraception	13.681	0.109	<0.001	−1810.03	28.01	0.000001	1211177.728
Polygyny	13.54	0.108	<0.001	−1809.78	28.26	0.000001	1369947.414
Wealth	12.04	0.097	<0.001	−1807.05	30.99	0.000000	5375749.886
Nutritionalstress	11.74	0.095	<0.001	−1806.51	31.54	0.000000	7045067.301
Motherage	3.845	0.033	0.023	−1791.53	46.51	0.000000	12573429647

Further evidence was provided by negative correlation of twenty four (of the twenty eight parasites considered) specific parasite loads with population SRB (Table 4). Similar patterns were observed when countries were grouped into tropical and temperate zones (Table 4). It must be noted that latitude failed to correlate with SRB in tropical (r = 0.031, n = 102, p = 0.753) and temperate (r = 0.180, n = 80, p = 0.111) zones.

**Table 4:** Correlations between individual parasite stress, latitude and sex ratio at birth in temperate and tropical countries.

	Latitude (*r, p*)	Offspring sex ratio		
		Total (*r, p*)	Tropical countries (*r, p*)	Temperate countries (*r, p*)
All parasites	−0.360, 0.000	−0.531, 0.000	−0.662, 0.000	−0.295, 0.008
1. Tuberculosis	−0.448, 0.000	−0.530, 0.000	−0.567, 0.000	−0.255, 0.023
2. STDs excluding HIV	−0.462, 0.000	−0.445, 0.000	−0.417, 0.000	−0.324, 0.003
2a. Syphilis	−0.335, 0.000	−0.341, 0.000	−0.333, 0.001	−0.293, 0.008
2b. Chlamydia	−0.658, 0.000	−0.526, 0.000	−0.499, 0.000	−0.251, 0.025
2c. Gonorrhea	−0.629, 0.000	−0.606, 0.000	−0.622, 0.000	−0.338, 0.002
3. HIV/AIDS	−0.185, 0.012	−0.395, 0.000	−0.426, 0.000	−0.310, 0.005
4. Diarrhoeal diseases	−0.474, 0.000	−0.490, 0.000	−0.464, 0.000	−0.309, 0.005
5. Childhood-cluster diseases	−0.405, 0.000	−0.435, 0.000	−0.430, 0.000	−0.259, 0.020
5a. Pertussis	−0.414, 0.000	−0.453, 0.000	−0.465, 0.000	−0.315, 0.004
5b. Poliomyelitis	−0.451, 0.000	−0.059, 0.428	0.390, 0.000	−0.136, 0.231
5c. Diphtheria	−0.191, 0.010	−0.328, 0.000	−0.368, 0.000	−0.117, 0.300
5d. Measles	−0.371, 0.000	−0.396, 0.000	−0.380, 0.000	−0.254, 0.023
5e. Tetanus	−0.343, 0.000	−0.366, 0.000	−0.386, 0.000	−0.115, 0.309
6. Meningitis	−0.267, 0.000	−0.233, 0.002	−0.150, 0.134	−0.146, 0.198
7a. Hepatitis B	−0.360, 0.000	−0.325, 0.000	−0.274, 0.005	−0.261, 0.020
7b. Hepatitis C	−0.289, 0.000	−0.295, 0.000	−0.299, 0.002	−0.120, 0.290
8. Malaria	−0.432, 0.000	−0.469, 0.000	−0.517, 0.000	0.075, 0.507
9. Tropical-cluster diseases	−0.519, 0.000	−0.520, 0.000	−0.520, 0.000	−0.358, 0.001
9a. Trypanosomiasis	−0.325, 0.000	−0.365, 0.000	−0.371, 0.000	−0.208, 0.064
9b. Chagas disease	−0.225, 0.002	−0.019, 0.500	0.218, 0.028	−0.179, 0.113
9c. Schistosomiasis	−0.405, 0.000	−0.515, 0.000	−0.547, 0.000	−0.319, 0.004
9d. Leishmaniasis	−0.350, 0.000	−0.333, 0.000	−0.276, 0.005	−0.193, 0.087
9e. lymphatic filariasis	−0.516, 0.000	−0.501, 0.000	−0.531, 0.000	−0.112, 0.325
9f. Onchocerciasis	−0.231, 0.002	−0.159, 0.032	−0.115, 0.249	Not applicable
10. Leprosy	−0.516, 0.000	−0.303, 0.000	−0.069, 0.491	−0.273, 0.014
11. Dengue	−0.154, 0.038	−0.073, 0.328	0.031, 0.754	−0.094, 0.048
12. Japanese encephalitis	−0.178, 0.016	0.037, 0.617	0.264, 0.007	−0.111, 0.327
13. Trachoma	−0.386, 0.000	−0.471, 0.000	−0.496, 0.000	−0.250, 0.025
14. Intestinal nematode infections	−0.615, 0.000	−0.465, 0.000	−0.321, 0.001	−0.300, 0.007
14a. Ascariasis	−0.455, 0.000	−0.408, 0.000	−0.339, 0.000	−0.175, 0.121
14b. Trichuriasis	−0.630, 0.000	−0.384, 0.000	−0.036, 0.721	−0.413, 0.000
14c. Hookworm disease	−0.649, 0.000	−0.485, 0.000	−0.326, 0.001	−0.363, 0.001

N = 182, 102 and 80 countries respectively for total, tropical zone and temperate zone.

## Discussion

The negative relationship between parasite stress and SRB was significant at the global level, and was repeated within tropical and temperate zones, even though these zones have distinct environmental conditions. Ridge regression showed that, polygyny, total fertility, son preference, latitude, and parasite stress are the significant predictors of human SRB. Model selection using AIC parameters suggest parasite stress is the best predictor among these significant variables. Interestingly, parasite stress could explain cross-cultural variation of SRB even better than son preference, which invariably results in higher SRB. Though the effects of latitude were similar to an earlier study [12], it must be noted that correlations of latitude and SRB with total parasite stress, and most of the individual parasite stress is almost similar in both magnitude and direction (Table 1 and Table 4). Since the correlation of latitude with SRB has no mechanistic physiological basis, it is possible that the latitudinal gradient of parasite (even at the level of individual parasites) stress is driving the association of SRB and latitude. SRB correlated strongly with total as well as most of the individual parasite stress levels within tropical and temperate regions, providing additional support for the relation between parasite stress and SRB. Indeed, earlier reports on the correlation of latitude with SRB have been contradictory, and have failed to provide causal basis [12], [35], [36].

Although most of the socioeconomic variables failed to predict SRB variation, it cannot be concluded that these variables are not involved. The correlation matrix shows that these variables are significantly interrelated with each other (Table S2), which might contribute to, or modify the effects of parasite stress on SRB. This potential source of endogeneity must be considered while interpreting the above results. It should also be noted that there may be unknown variables not included in this analysis that could drive the global variation of SRB. For example, ambient temperature [13], geography [37], environmental stressors [38], [39], and their interactions [7] proposed to influence SRB variation in large human populations, are also likely to play role in the cross-cultural variation of SRB. It is therefore, necessary to establish the extent to which such factors contribute to SRB variation by conducting further studies using world-wide data.

Sex allocation theory proposes that parental ability to select the offspring sex is responsive to factors that alter the relative importance of benefits and costs associated with choosing offspring sex [5], [40]. Theoretically, a male offspring is associated with higher benefits, as it can sire higher number of grandchildren as compared to a female offspring. These benefits depend on the quality of the male offspring; such that, a high-quality male can produce a large number of grandchildren whereas a poor-quality male may never get an opportunity to father a child due to its inability to acquire a mate. Nevertheless, the chances of reproduction in female sex do not vary so much with her quality, as most of them breed irrespective of quality. Hence, to achieve maximum returns, parents in good condition will be expected to produce more sons whereas parents in poor condition will be benefited by biasing investment in daughters. The direct relationship between parasite stress and SRB found in my sample may suggest that women do select daughters where costs of bearing sons are difficult to meet (e.g. in underdeveloped countries with high poverty and poor healthcare). Such parental selection of offspring sex may significantly contribute to cross-cultural variation in SRB. However, these results cannot imply that the SRB adjustments across cultures follow Trivers and Willard’s predictions, because majority of the human populations are monogamous.

That SRB is better predicted by the parasite stress than by total fertility, polygyny, son preference, and mortality rates suggest that cross-cultural variation in offspring sex is not mediated solely by variation in life-history traits and mating strategies. This is important, as life history traits were believed to have strong influence on offspring sex ratio [10], [28], [41]. Indeed, in my sample, wealth, which positively select for sons, also showed a weaker correlation with SRB as compared to parasite stress. Mace et al. noted that low birth sex ratio within African populations is correlated with fertility [23]. They suggested that this may be genetically determined, as black Americans living in the USA also have SRB values similar to that of most African populations [42]. However, in my analysis, parasite stress correlated stronger than total fertility with SRB, across the world. Human populations with higher wealth and adequate healthcare facilities have lower fertility rates and better healthcare facilities, which suggest that, with improvement in socioeconomic condition brought-about by growth of wealth; variables like fertility and parasite stress could show significant improvements. Hence, it is unlikely that the relation between SRB and fertility is solely mediated by genetic factors, as suggested by Mace et al. (2003). Ample unequivocal empirical evidence shows that black Americans are more affected by infectious and nutritional disorders and are less healthy than white Americans [43]–[50]. This strongly suggest that similar to native African populations, black Americans could be inclined to favor more female births owing to subnormal body condition but not genetic predisposition.

The possible physiological basis for the relation of SRB and parasite stress could be derived from the sex differences in the costs of offspring production. Male fetus grows faster [51], and requires significantly higher parental investment during gestation [52], which means that, to produce sons, women should be in superior body condition to meet higher physiological costs required. Indeed, male fetuses are more spontaneously aborted than a female fetus due to nutritional deficiencies and exposure to environmental toxicants [2], [53]. It is therefore, possible that, various factors that influence maternal investment ability (parasite stress in the present analysis) are more likely than genetic and geographical factors to form the basis for striking cross-cultural variation in SRB, without any adaptive significance. However, my analysis still fails to rule out the role of genetic factors, but provide a strong support for the relation between condition and sex ratio. Alternatively, some studies have shown a lack of association between parental condition (measured by physical indices) and offspring sex ratio [54]–[57], which means that, parasite stress may have direct effects on SRB, if parental condition is not influencing SRB variation across cultures.

There are reports of association of certain infections with altered SRB in humans. Where hepatitis-B and toxoplasmosis infection was associated with excess son births [58]–[61], cytomegalovirus and vericella seropositivity was associated with excess daughters [62]–[64]. However, it must be noted that these studies were conducted on infected individuals, whereas my analysis has considered the parasitic load as a proxy of condition for large populations. Further, the role of hepatitis-B has been questioned by a recent re-analysis [65], whereas toxoplasmosis is believed to increase male births by various effects like relaxing the stringency of quality control during embryogenesis, producing immunosuppression, to increase spread, etc [58]. Hence, care must be taken before extrapolating my results in the light of these studies.

Although my results support the predictions of parasite stress hypothesis, further studies must be done to establish causation. Longitudinal studies designed to monitor the parasite stress in an individual from childhood, and its effects on SRB could provide causal support for the present statistical findings.

## Supporting Information

Table S1 **Descriptive statistics of the study variables.**
(DOCX)

Table S2 **Correlation matrix of the study variables.** Values below the diagonal are sample sizes (number of countries), values above the diagonal are correlation coefficients, followed by respective two tailed p values.
(DOCX)
