# Supplementary material for: Parasite Stress Predicts Offspring Sex Ratio
Source: PLoS One. 2012 Sep 26;7(9):e46169. doi: 10.1371/journal.pone.0046169 (PMC3458865; doi:10.1371/journal.pone.0046169)
Supplement: Table S1 — Descriptive statistics of the study variables. (DOCX) [file pone.0046169.s001.docx]

Table S1. Descriptive statistics of the study variables.

|  | **N** | **Minimum** | **Maximum** | **Mean** | **Std. Deviation** |
| --- | --- | --- | --- | --- | --- |
| Parasite stress | 192 | 2.17 | 4.81 | 3.33 | 0.72 |
| Contraception | 179 | 3.00 | 89.00 | 48.60 | 22.36 |
| Polygyny | 122 | 0.00 | 1.00 | 0.44 | 0.40 |
| Son preference | 121 | 0.00 | 1.00 | 0.14 | 0.24 |
| Total fertility | 198 | 0.95 | 7.12 | 2.87 | 1.45 |
| Wealth | 179 | 2.46 | 4.83 | 3.82 | 0.55 |
| Mother age | 195 | 22.00 | 32.00 | 26.08 | 2.99 |
| Latitude | 247 | 0.00 | 78.00 | 25.92 | 17.16 |
| Health adjusted life expectancy | 193 | 35.00 | 76.00 | 60.05 | 9.70 |
| Adult mortality rate | 187 | 53.00 | 772.00 | 210.20 | 129.14 |
| Maternal mortality ratio | 172 | 2.00 | 1400.00 | 210.10 | 287.24 |
| Under-five mortality rate | 192 | 1.70 | 209.00 | 45.21 | 51.28 |
| Infant mortality rate | 192 | 0.90 | 133.70 | 31.82 | 31.45 |
| Sex ratio at birth | 226 | 1.01 | 1.13 | 1.05 | 0.02 |
