# Supplementary material for: Parasite Stress Predicts Offspring Sex Ratio
Source: PLoS One. 2012 Sep 26;7(9):e46169. doi: 10.1371/journal.pone.0046169 (PMC3458865; doi:10.1371/journal.pone.0046169)
Supplement: Table S2 — Correlation matrix of the study variables. Values below the diagonal are sample sizes (number of countries), values above the diagonal are correlation coefficients, followed by respective two tailed p values. (DOCX) [file pone.0046169.s002.docx]

Table S2. Correlation matrix of the study variables. Values below the diagonal are sample sizes (number of countries), values above the diagonal are correlation coefficients, followed by respective two tailed p values.

|  | **1** | **2** | **3** | **4** | **5** | **6** | **7** | **8** | **9** | **10** | **11** | **12** | **13** | **14** |
| --- | --- | --- | --- | --- | --- | --- | --- | --- | --- | --- | --- | --- | --- | --- |
| **Sex ratio at birth** |  | -0.523, 0.000 | 0.353,  0.000 | -0.388, 0.000 | 0.340, 0.000 | -0.444, 0.000 | 0.359, 0.000 | 0.062, 0.401, | 0.370, 0.000 | 0.463, 0.000 | -0.469, 0.000 | -0.451, 0.000 | -0.439, 0.000 | -0.425, 0.000 |
| **Parasite stress** | 190 |  | -0.652,  0.000 | 0.580, 0.000 | -0.177, 0.056 | 0.799, 0.000 | -0.829, 0.000 | -0.369, 0.000 | -0.693, 0.000 | -0.934, 0.000 | 0.867, 0.000 | 0.775, 0.000 | 0.820, 0.000 | 0.848, 0.000 |
| **Contraception** | 177 | 176 |  | -0.602, 0.000 | -0.030, 0.748 | -0.766, 0.000 | 0.70, 0.000 | 0.006,  0.936 | 0.501, 0.000 | 0.699, 0.000 | -0.546, 0.000 | -0.708, 0.000 | -0.732, 0.000 | -0.735, 0.000 |
| **Polygyny** | 119 | 117 | 115 |  | 0.135, 0.140 | 0.607, 0.000 | -0.402, 0.000 | 0.214,  0.019 | -0.304, 0.001 | -0.642, 0.000 | 0.565, 0.000 | 0.595, 0.000 | 0.630, 0.000 | 0.612, 0.000 |
| **Son preference** | 119 | 117 | 115 | 121 |  | -0.035, 0.703 | 0.088, 0.361 | 0.260,  0.004 | 0.210, 0.022 | 0.109, 0.243 | -0.190, 0.041 | -0.051, 0.586 | -0.082, 0.379 | -0.056, 0.548 |
| **Total fertility** | 195 | 180 | 168 | 119 | 119 |  | -0.795, 0.000 | -0.178, 0.014 | -0.564, 0.000 | -0.833, 0.000 | 0.704, 0.000 | 0.841, 0.000 | 0.875, 0.000 | 0.869, 0.000 |
| **GNI** | 178 | 174 | 165 | 111 | 111 | 173 |  | 0.362,  0.000 | 0.582, 0.000 | 0.843, 0.000 | -0.722, 0.000 | -0.761, 0.000 | -0.784, 0.000 | -0.816, 0.000 |
| **Mother age** | 188 | 176 | 169 | 120 | 120 | 188 | 173 |  | 0.262, 0.000 | 0.336, 0.000 | -0.347, 0.000 | -0.179, 0.000 | -0.194, 0.009 | -0.208, 0.005 |
| **Latitude** | 224 | 191 | 178 | 119 | 119 | 198 | 178 | 191 |  | 0.565, 0.000 | -0.435, 0.000 | -0.509, 0.000 | -0.512, 0.000 | -0.535, 0.000 |
| **Health adjusted life expectancy** | 191 | 190 | 178 | 117 | 117 | 180 | 172 | 179 | 180 |  | -0.929, 0.000 | -0.853, 0.000 | -0.898, 0.000 | -0.915, 0.000 |
| **Adult mortality rate** | 186 | 184 | 173 | 116 | 116 | 179 | 176 | 178 | 186 | 179 |  | 0.791, 0.000 | 0.789, 0.000 | 0.800, 0.000 |
| **Maternal mortality ratio** | 171 | 168 | 160 | 118 | 118 | 171 | 175 | 178 | 171 | 170 | 170 |  | 0.925, 0.000 | 0.911, 0.000 |
| **Under-five mortality rate** | 191 | 187 | 175 | 117 | 117 | 183 | 177 | 178 | 191 | 180 | 187 | 171 |  | 0.989, 0.000 |
| **Infant mortality rate** | 191 | 187 | 175 | 117 | 117 | 183 | 177 | 178 | 191 | 180 | 1870 | 171 | 192 |  |
